# “Live high‐train low” induced changes in hemoglobin mass and the erythropoietin‐erythroferrone‐hepcidin axis in female endurance athletes

**DOI:** 10.14814/phy2.70707

**Published:** 2025-12-19

**Authors:** Titta Kuorelahti, Johanna K. Ihalainen, Vesa Linnamo, Claire Badenhorst, Oona Kettunen, Ritva Mikkonen

**Affiliations:** ^1^ Sports Technology Unit Vuokatti, Faculty of Sport and Health Sciences University of Jyväskylä Vuokatti Finland; ^2^ Finnish Institute of High Performance Sport KIHU Jyväskylä Finland; ^3^ East Metropolitan Health Service, Royal Perth Perth Western Australia Australia; ^4^ School of Medical and Health Science Edith Cowan University Perth Western Australia Australia

**Keywords:** erythroferrone, hemoglobin mass, hepcidin, iron status, normobaric hypoxia

## Abstract

The effects of a 21‐day live high‐train low (LHTL) on hemoglobin mass (Hb_mass_) and iron demand including the erythropoietin (EPO)‐erythroferrone (ERFE)‐hepcidin axis and routine iron markers were investigated. Fifteen female endurance athletes completed either 21‐day LHTL in normobaric hypoxia (2500 m, ~18 h·day^−1^) (INT, *n* = 8) or lived and trained in normoxia (CON, *n* = 7). Hb_mass_ and resting blood were collected before and after the intervention. An additional blood sample was collected on Day 6 for INT. 21‐day LHTL increased Hb_mass_ 3.8% in INT (*p* < 0.001). EPO increased 35.6% from Day 0 to Day 6 in INT (*p* = 0.037) and then decreased 42.4% from Day 6 to Day 21 (*p* = 0.019). In INT, no changes were detected in ERFE or hepcidin, and from the routine iron markers only serum transferrin receptor increased from Day 0 to Day 21 (13.9%, *p* = 0.013). In CON, no changes were detected in Hb_mass_ or iron markers. In INT, Hb_mass_ and ferritin were positively associated (Day 0 to Day 6, *p* = 0.005). Thus, hepcidin and ERFE may not provide additional information regarding iron demand following 6‐ or 21‐day LHTL compared to routine iron markers. The relationship between Hb_mass_ and ferritin indicates that adequate ferritin levels are needed during hypoxia to support hematological adaptations.

## 1 INTRODUCTION

Altitude training in its many forms has become a common part of many elite athletes' annual training program due to its potential to improve altitude and sea level performance. In the majority of studies, performance improvements have been linked to the hematological adaptations that occur due to hypoxia: prolonged hypoxic exposure increases erythropoietic activity, which leads to increased red blood cell production and, thereby, oxygen‐carrying capacity (Saunders et al., 2013; Schmidt & Prommer, 2010). Typically, a 3%–5% change in hemoglobin mass (Hb_mass_) has been reported following 3–4 weeks of hypoxic training in natural and simulated (e.g., hypoxic apartments) moderate altitude (Garvican et al., 2012; Kettunen et al., 2023) although substantial variability in the adaptation exists both within and between subjects (Hauser et al., 2017; Nummela et al., 2021).

Supporting Hb_mass_ adaptations requires sufficient iron availability as the transition from normoxia to hypoxia increases erythropoietic iron demand three‐ to five‐fold (Reynafarje et al., 1959). To meet iron requirements in hypoxic environments, changes occur in the expression of the master iron regulator serum hepcidin (Piperno et al., 2011). Hepcidin controls iron efflux to circulation from duodenal enterocytes and iron‐recycling macrophages by internalizing and degrading ferroportin, the sole known cellular iron exporter (Nemeth et al., 2004). Following a 2‐day exposure to hypoxia, hepcidin expression is suppressed due to a rapid increase in kidney erythropoietin (EPO) synthesis, which leads to the prompted erythroid production of a hepcidin‐inhibiting hormone erythroferrone (ERFE) (Govus et al., 2017; Kautz et al., 2014; Robach et al., 2020). In addition, increased iron demand in hypoxia may elevate plasma transferrin and soluble transferrin receptor (sTfR) levels to support sufficient iron supply for bone marrow erythropoiesis (Piperno et al., 2011; Robach et al., 2004; Wehrlin et al., 2006) as well as increase mobilization of iron from iron stores, thereby leading to reduced serum ferritin levels (Mckay et al., 2024; Nummela et al., 2021).

Investigating the changes in iron regulatory markers ERFE and hepcidin has recently been suggested to serve as a valuable approach for assessing alterations in iron demand. For example, Breenfeldt Andersen et al. (2021) observed that both ERFE and hepcidin quickly respond to 3 weeks of recombinant human EPO treatment in healthy subjects. The authors also reported a significant increase in ERFE after 1 week altitude residence (2320 m) that continued through to weeks 2 and 3 in altitude, while no changes in serum hepcidin occurred (Breenfeldt Andersen et al., 2021). Similar observations in athletic populations were made by Płoszczyca et al. (2023) who found suppressed hepcidin and ERFE following 3 weeks of live high‐train low exposure (LHTL) in trained male cyclists, and Mckay et al. (2024) who found significant reduction in hepcidin but not in ERFE following 7 days of altitude exposure (1800 m) in male runners.

To address the increased demand for iron in hypoxia, the current practical recommendations for athletes include ensuring sufficient iron stores (serum ferritin >30–50 μg·L^−1^) as well as iron supplementation prior to and during altitude training camps (Clénin et al., 2015; Stellingwerff et al., 2019). However, this iron sufficiency has not robustly translated into a positive Hb_mass_ response, highlighting the need for a deeper understanding of changes in iron demand and regulation during hypoxic training periods (Koivisto‐Mørk et al., 2021; Nummela et al., 2021). To our knowledge, the hypoxia‐induced changes in iron regulatory markers ERFE and hepcidin in highly trained athletes have only been investigated in two studies (Garvican‐Lewis et al., 2018; Mckay et al., 2024), while none have focused solely on females. Even during sea level training, female athletes tend to face difficulties in meeting the elevated iron requirements resulting from exercise and increased iron loss through menstruation (Harvey et al., 2005), thus placing them at an increased risk for iron deficiency (ferritin <35 μg·L^−1^) and its progression to iron deficiency anemia (ferritin <12 μg·L^−1^ and hemoglobin <115 g·L^−1^) (Clénin et al., 2015; Peeling et al., 2007). The possible differences in iron requirements warrant a female‐specific approach to properly address the distinct hypoxia‐induced changes in iron demand. As such, the aim of this study was to assess the changes in iron regulation during a 21‐day live high‐train low intervention in female endurance athletes by determining changes in the EPO‐ERFE‐hepcidin axis. In addition, the study evaluated whether the changes in iron regulatory pathways and routine iron markers were associated with changes in Hb_mass_.

## 2 MATERIALS AND METHODS

### 2.1 Participants

This study recruited 15 national to international level (Tier 3 to 4) female cross‐country skiers, biathletes, and ski‐orienteers (age 23 ± 4), with each participant self‐selecting into either intervention group (INT, *n* = 8) or control group (CON, *n* = 7) (McKay et al., 2022). During the measurements, none of the participants presented any signs of inflammation or illness. The participants were non‐anemic, but stage 1 iron deficiency was observed in five participants in INT and three in CON (ferritin 20–35 μg·L^−1^, hemoglobin concentration >115 g·L^−1^, and transferrin <16%) (Peeling et al., 2007). All participants were fully informed of the experimental procedures and provided written consent to participate. The ethical committee of the University of Jyväskylä approved the study (29/13.00.04.00/2021, January 21, 2021), and all the measurements were conducted in accordance with the Declaration of Helsinki except for pre‐registration in a database.

### 2.2 Study design

The study was carried out during the athletes' preparatory season from late July to mid September 2023. During the study, INT completed a 21‐day LHTL period in hypoxic apartments of the Olympic Training Center (Vuokatti Sport), which were set to simulate an altitude of 2500 m (Fraction of inspired oxygen, FIO_2_ = 15.8%) and were monitored with Dräger Pac8500 (Dräger, Germany). For the same duration (21 days), CON both lived and trained in normoxia. During the intervention, INT spent approximately 17.8 ± 1.3 h·day^−1^ in hypoxia with a corresponding hypoxic dose of 930 km·h (Garvican‐Lewis et al., 2016), ∼150 m above sea level.

Hb_mass_, fasted venous blood samples, and body composition and mass (Inbody 770, Biospace Co., Seoul, Korea) were assessed in all participants 1–2 days before and after the 21‐day intervention. To investigate short‐term changes to LHTL in blood biomarkers, an additional fasted venous blood sample was collected from INT on the 6th morning in normobaric hypoxia (time spent in hypoxia: 19.7 ± 0.8 h·day^−1^, corresponding hypoxic dose = 280 km·h). All measurements were conducted at the same time of day to avoid the influence of circadian variation. The participants were instructed to refrain from caffeine, alcohol, nicotine, and antihistamines 24 h before arriving for morning blood samples and not to exercise 12 h prior to measurements.

During the study, iron supplementation was monitored but not controlled. In INT, five participants supplemented with iron according to recommendations from their own doctors or coaches. The approximate elemental intake was 33 ± 43 mg·day^−1^ (20–200 mg·day^−1^). In CON, none of the participants consumed iron supplements. Full details of the supplemental iron intake of these athletes have been presented in our previous publication (Kuorelahti et al., 2025). Menstrual cycle was not monitored during the study, as the study protocol did not allow standardized scheduling of test sessions around the menstrual cycle. The use of hormonal contraceptives, in turn, was monitored but not controlled; however, all hormonal contraceptive‐using participants used progestin‐only contraceptive methods. In INT, four participants used intrauterine progestin‐only contraception while four did not employ any form of contraception. In CON, one participant used intrauterine progestin‐only contraception, two were on progestin‐only pills, and four were not on any form of contraception.

### 2.3 Hemoglobin mass measurement

The optimized carbon monoxide rebreathing method (Schmidt & Prommer, 2005) and SpiCo Calculation Software 2.2 (Blood tec GmbH, Bayreuth, Germany) were used to measure Hb_mass_. The procedure included 2 min of rebreathing of O_2_‐CO_2_ gas mixture (O_2_∼3L; CO_2_ 0.9 mL·kg^−1^) via a closed‐circuit spirometer (SpiCO, Blood tec). The fraction of carboxyhemoglobin (%HbCO) was measured with capillary blood samples that were drawn from the fingertip three times at baseline and twice at 6 and 8 min after the rebreathing procedure using the ABL90 FLEX blood gas analyzer (Radiometer Medical ApS). The changes in %HbCO were used to calculate the Hb_mass_. A typical error reported for the method is 1.7% (Schmidt & Prommer, 2005).

### 2.4 Venous blood samples

All venous blood samples were collected at 7–8 am in a fasted state from an antecubital vein. The blood was drawn into two 6 mL Vacuette serum clot activator tubes (Greiner‐Bio‐One GmbH) and a 4 mL EDTA gel serum tube. Immediately after the collection, the EDTA tubes were refrigerated and later transported to Vita laboratories for basic blood count analysis. The samples were analyzed within 36 h from the obtainment with Sysmex XN‐1000 (SysmexCo.).

The serum samples clotted for 30 min before centrifugation at 1825 × *g* for 10 min. After centrifugation the serum was transferred into aliquots and stored first at −20°C and then at −80°C until further analysis. For the analysis of sTfR, transferrin, serum iron, and EPO, frozen serum was taken to Vita laboratories (Helsinki, Finland) where Roche Cobas Pro c503 analyzer (Roche Diagnostics, Switzerland) was used. sTfR was analyzed using particle‐enhanced immunoturbidimetric assay (Cat. No. ACN 21060), transferrin with immunoturbidimetric assay (Cat. No. ACN 21150), and serum iron with colorimetric assay (Cat. No. ACN 20770). EPO, as well as serum ferritin, high‐sensitivity C‐reactive protein (hs‐CRP), and interleukin‐6 (IL‐6) were analyzed using the Immulite 2000 and immunoassay kits (Immulite, Siemens, IL, USA; Cat. Nos. L2KEPN2, L2KFE2, L2KCRP2, and L2K6P2). Circulating hepcidin and ERFE were analyzed with enzyme‐linked immunosorbent assay with commercial reagents (Quantikine human hepcidin ELISA, R&D Systems Inc., Minneapolis, MN, USA; Erythroferrone (human) ELISA kit, AdipoGen Life Sciences, Epalinges, Switzerland). The detection limits and inter‐assay coefficients of variation were 0.1 g/L and 0.8% for transferrin, 0.5 mg·L^−1^ and 3.4% for sTfR, 0.9 μmol·L^−1^ and 2.6% for serum iron, 1 U·L^−1^ and 7.1% for EPO, 0.1 mg·L^−1^ and 8.3% for hs‐CRP, 2.0 pg·mL^−1^ and 5.3% for IL‐6, 0.4 μg·L^−1^ and 12.1% for serum ferritin, 3.8 pg·mL^−1^ and 8.0% for hepcidin, and 270 pg·mL^−1^ and 3.69% for ERFE.

### 2.5 Training monitoring

All participants followed their normal training regimen throughout the study. Training volume and intensity distribution were monitored and data collected and stored online via Polar GPS‐enabled watches (Polar Flow, Polar Electro Oy, Kempele, Finland). The monitoring started 3 weeks before and continued throughout the 21‐day intervention. The training was divided into four categories based on the exercise heart rate distribution: low‐intensity training (LIT, target blood lactate <2.5 mmol·L^−1^), moderate‐intensity training (MIT, target blood lactate 2.5–4.0 mmol·L^−1^), high‐intensity training (HIT, target blood lactate 4.0–10.0 mmol·L^−1^) (Sandbakk & Holmberg, 2017), and strength training.

### 2.6 Statistical analyses

Statistical analyses were performed using SPSS Statistics 28 (IMB). Shapiro–Wilk test was used to assess the assumption of normality, and non‐normally distributed data was log‐transformed (hs‐CRP, IL‐6). Repeated measures ANOVA was used to investigate the effect of time (Day 0, Day 6, and Day 21) on blood biomarkers in INT. In addition, mixed ANOVA with time (Day 0 and Day 21) as a within‐subject factor and group (INT, CON) as a between‐subject factor was used to analyze the changes and between‐group differences in blood biomarkers as well as training volume and intensity. If the sphericity assumption was violated, the Greenhouse–Geisser correction was applied. Post hoc Bonferroni correction was applied to identify the specific differences between trials. Possible changes in training volume or intensity distribution a week before each measurement point (Day 0, Day 6, and Day 21) as well as iron supplementation and hormonal contraceptive use were used as covariates to detect whether they influenced the responses Hb_mass_ or circulatory iron markers. The Pearson correlation coefficient was used to describe associations between changes in Hb_mass_ and iron markers. Associations were also investigated between the EPO‐ERFE‐hepcidin axis and serum ferritin to see whether the pre‐hypoxic iron levels or the changes in serum ferritin levels affected the changes in iron regulation in hypoxia. The level of significance was set at *p* < 0.05 and the results are presented as mean and standard deviation (± SD).

## 3 RESULTS

### 3.1 Hemoglobin mass

Table 1 presents the overall means in Hb_mass_ and all blood biomarkers. A significant main effect of time (*F* = 13.5, *p* = 0.003) and time × group interaction (F = 10.2, *p* = 0.007) were observed in Hb_mass_. The post hoc analysis revealed that in INT Hb_mass_ increased by 3.8 ± 2.0% from Day 0 to Day 21 (*p* < 0.001), while in CON, no changes were detected (0.4 ± 2.2%, *p* = 0.750).

**TABLE 1:** Hb_mass_ and iron markers before, during, and after 21 days of LHTL or normoxia.

	INT			CON		Mixed ANOVA			Repeated measures ANOVA
	Day 0	Day 6	Day 21	Day 0	Day 21	Time	Group	Group × time	Time
Hb_mass_ (g)	656 ± 60		681 ± 64**	627 ± 62	629 ± 55	***p* = 0.003**	*p* = 0.212	***p* = 0.007**	
Ferritin (μg·L^−1^)	39.6 ± 19.6	38.9 ± 23.7	34.3 ± 16.3	54.9 ± 31.5	47.1 ± 24.4	***p* = 0.044**	*p* = 0.250	*p* = 0.657	*p* = 0.335
sTfR (mg·L^−1^)	2.59 ± 0.42	2.83 ± 0.41	2.91 ± 0.43*	2.59 ± 0.37	2.50 ± 0.31	*p* = 0.171	*p* = 0.276	***p* = 0.027**	*p* = 0.078
Ferritin index	1.69 ± 0.36	1.88 ± 0.36	1.99 ± 0.50	1.55 ± 0.27	1.58 ± 0.36	*p* = 0.053	*p* = 0.157	*p* = 0.094	*p* = 0.074
TSAT (%)	28.9 ± 7.1	28.6 ± 9.8	24.1 ± 7.6	24.9 ± 10.3	26.6 ± 14.0	*p* = 0.635	*p* = 0.850	*p* = 0.320	*p* = 0.499
Transferrin (g·L^−1^)	2.30 ± 0.24	2.35 ± 0.24	2.34 ± 0.23	2.46 ± 0.21	2.47 ± 0.21	*p* = 0.527	*p* = 0.775	*p* = 0.775	*p* = 0.648
Iron (μmol·L^−1^)	17.5 ± 5.0	17.8 ± 7.0	14.8 ± 4.6	15.9 ± 7.1	17.6 ± 10.2	*p* = 0.823	*p* = 0.835	*p* = 0.343	*p* = 0.549

Abbreviations: Hb_mass_, hemoglobin mass; sTfR, soluble transferrin receptor; TSAT, transferrin saturation.

* Significantly different from Day 0 *p* < 0.05.

** *p* < 0.001.

### 3.2 EPO‐ERFE‐hepcidin‐axis

**FIGURE 1:**
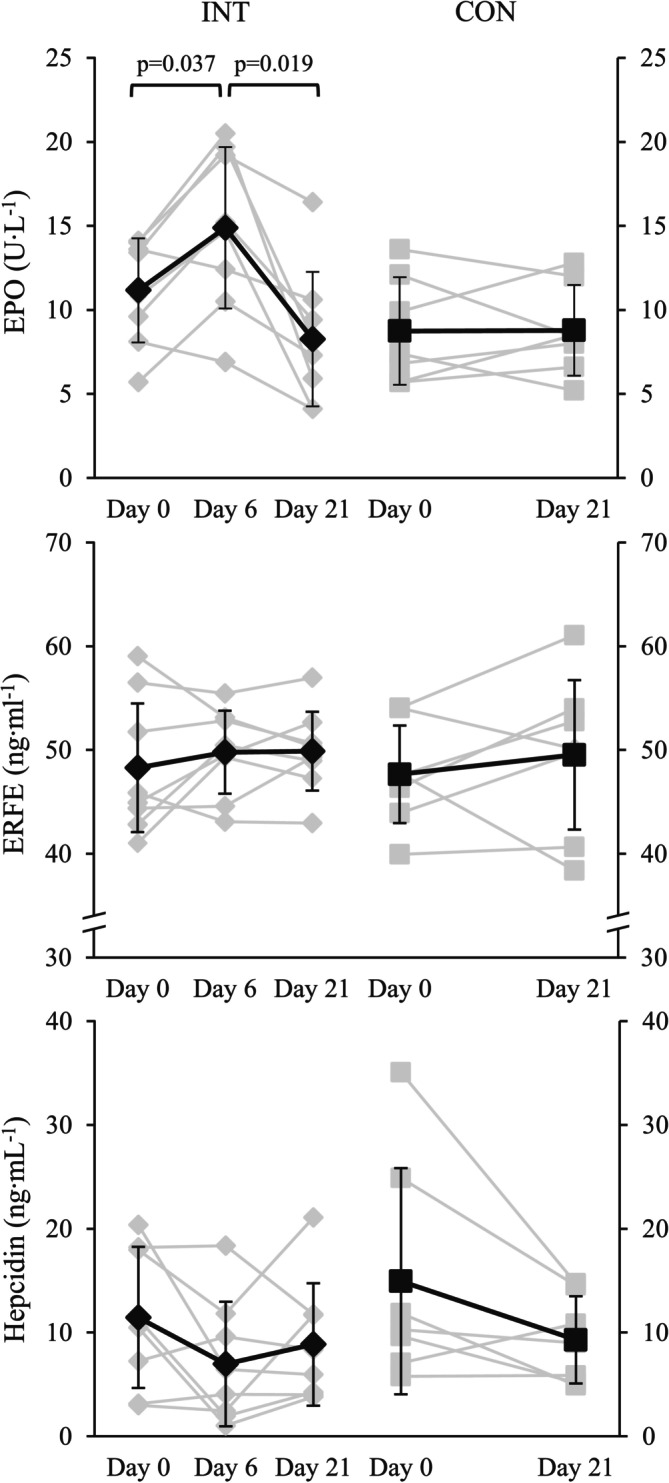
EPO, ERFE, and hepcidin before and after 6, and 21 days of LHTL (INT) or living and training in normoxia (CON). The black line represents group means (± SD) across time points and gray lines individual participants.

Figure 1 presents changes in EPO, ERFE, and hepcidin from Day 0 to Day 6 and Day 21 in both groups. In the repeated measures ANOVA analysis, a significant effect of time on EPO levels was observed (*F* = 10.9, *p* = 0.001) in INT. The initial increase in EPO from Day 0 to Day 6 was 35.6% (*p* = 0.037), after which the levels declined by 42.4% to Day 21 (*p* = 0.019; −23.4% from Day 0). In CON, no changes in EPO levels were detected. In ERFE or hepcidin, no changes were detected regardless of the group.

### 3.3 Routine iron biomarkers

A significant time × group interaction was observed in sTfR (*F* = 6.2, *p* = 0.027) in mixed ANOVA analysis. In INT, sTfR increased 13.9% (*p* = 0.013) from Day 0 to Day 21 while no significant changes were observed in CON (−5.7%, *p* = 0.490). A significant main effect of time in serum ferritin was detected (*F* = 5.0, *p* = 0.044), but in the post hoc analysis, no changes or between‐group differences were found. The other routine iron markers, including transferrin, transferrin saturation, ferritin index, and serum iron, exhibited no changes over time or between groups.

### 3.4 Associations between variables

**FIGURE 2:**
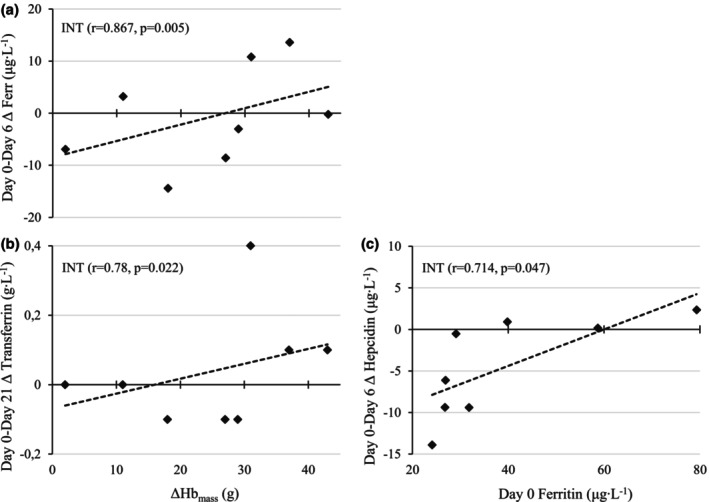
Associations between changes in Hb_mass_ and (a) Day 0 to Day 6 changes in ferritin, (b) Day 0 to Day 21 changes in transferrin, and (c) baseline ferritin and Day 0 to Day 6 changes in hepcidin in INT.

The changes in Hb_mass_ were not associated with changes in ERFE or hepcidin following 6‐ or 21‐day LHTL in INT. In turn, a smaller reduction in ferritin during the first 6 days of LHTL was associated with a higher increase in Hb_mass_ in INT (*r* = 0.87, *p* = 0.005) (Figure 2), while the changes from Day 0 to Day 21 or pre‐hypoxic ferritin levels were not associated with Hb_mass_ adaptations. In addition, the changes in Hb_mass_ were negatively associated with changes in EPO (*r* = −0.82, *p* = 0.012) and positively associated with changes in transferrin (*r* = 0.782, *p* = 0.022) following the 21‐day LHTL in INT. In CON, the changes in Hb_mass_ were not associated with changes in any of the iron markers.

A positive association between pre‐hypoxic ferritin and Day 0 to Day 6 change in hepcidin in hypoxia was observed in INT (*r* = 0.714, *p* = 0.047), showing that lower ferritin levels at baseline were associated with a greater reduction in hepcidin following the first 6 days of LHTL (Figure 2). In addition, a positive association between the changes in EPO and ferritin from Day 0 to Day 21 was observed in INT (*r* = 0.884, *p* = 0.004). In CON, the Day 0 to Day 21 changes in hepcidin levels were positively associated with baseline ferritin levels (*r* = 0.81, *p* = 0.028) as well as with the Day 0 to Day 21 changes in ferritin (*r* = −0.90, *p* = 0.006).

### 3.5 Training, inflammation, and the effects of iron supplementation and hormonal contraceptive use

The mean training volume during the monitoring period was 17.6 ± 1.9 h·week^−1^ (LIT 84.5 ± 3.6%, MIT 6.0 ± 2.0%, HIT 1.2 ± 1.3%, ST 8.4 ± 3.0%) in INT and 16.2 ± 2.4 h·week^−1^ (LIT 83.5 ± 4.9%, MIT 7.2 ± 3.6%, HIT 1.2 ± 0.8%, ST 8.1 ± 2.4%) in CON. There were no changes in weekly training volume or the proportion of LIT, MIT, and strength training prior to Day 0, Day 6, and Day 21 in either group. The proportion of HIT increased in INT from Day 0 to Day 21 (0.5 ± 0.8% to 2.6 ± 2.6%, *p* = 0.015), but when tested as a covariate, the change did not appear to affect changes in iron markers. No changes were observed in hs‐CRP or IL‐6 in either group at any time point. Finally, neither iron supplementation nor hormonal contraceptive use appeared to influence changes in iron markers when included as covariates.

## 4 DISCUSSION

This study investigated changes in iron demand and regulation during a 21‐day LHTL intervention in Tier 3 to 4 level female endurance athletes by investigating changes in the EPO‐ERFE‐hepcidin axis and several routine iron markers (McKay et al., 2022). No marked changes were detected in hepcidin and ERFE following 6‐ or 21‐day prolonged hypoxic exposure, suggesting that in isolation these iron regulatory markers may not provide additional information regarding changes in iron demand and regulation compared to routine iron markers ferritin, transferrin, TSAT, and sTfR. In addition, the study examined whether the changes in iron markers were associated with changes in Hb_mass_ and found a positive association between Hb_mass_ and serum ferritin following 6 days in hypoxia in INT. This observation suggests that maintaining serum ferritin levels prior to and during acute LHTL exposure may support the intended hematological adaptations.

### 4.1 Hb_mass_ and EPO‐ERFE‐hepcidin axis

In hypoxia, regulation of iron metabolism is altered due to the rapid increase in renal and liver EPO production simulated by the hypoxic stabilization of the hypoxia‐inducible factor‐2α (Haase, 2013). During sustained hypoxic exposures, EPO levels tend to spike within 48 h, followed by a decline to near sea levels over a 12‐day timeframe (Garvican et al., 2012). Furthermore, after the cessation of hypoxic stimulus, EPO levels may temporarily drop below sea level baseline values (Garvican et al., 2012). Our results are in line with the previous observations as EPO in INT increased by 36% from Day 0 to Day 6 while after the 21‐day LHTL the levels were 23% lower compared to Day 0. In CON no changes in EPO were detected from Day 0 to Day 21. The initial increase in EPO in INT suggests that the hypoxic stimulus during the first 6 days of LHTL was sufficient to increase erythropoietic activity, which typically leads to accelerated red blood cell production during prolonged (3–4 week) hypoxic exposures (Garvican et al., 2012; Kettunen et al., 2023; Lundby et al., 2012). Indeed, a 3.8% ± 2.0% increase in Hb_mass_ was observed from Day 0 to Day 21 in INT with the change aligning with Garvican‐Lewis et al. (2016) exponential model prediction, designed to estimate the change in Hb_mass_ relative to accumulate hypoxic exposure, as well as with the Hb_mass_ changes observed in previous studies using a similar hypoxic dose (Garvican‐Lewis et al., 2018; Kettunen et al., 2023; Koivisto‐Mørk et al., 2021). In CON no changes in Hb_mass_ were detected, as expected.

The typical changes in EPO levels and Hb_mass_ in INT suggest increased erythroid activity, which research has noted may require sufficient iron availability to ensure adequate iron delivery to bone marrow for red blood cell production. Stimulated by the increase in EPO levels, ERFE and hepcidin have been suggested to serve as sensitive markers for detecting increased erythropoietic activity (Breenfeldt Andersen et al., 2021). In the present study, however, no changes were observed in either marker after 6 or 21 days of LHTL. In addition, these changes were not associated with changes in Hb_mass_. While ERFE appears to sensitively respond to recombinant EPO treatment (Breenfeldt Andersen et al., 2021; Robach et al., 2020), the current evidence on the effects of sustained moderate hypoxia on ERFE in athletes is limited and inconsistent. Although Breenfeldt Andersen et al. (2021) found a ~2‐fold increase in ERFE following one‐week altitude exposure at 2320 m in healthy males and females, Mckay et al. (2024) observed no changes in ERFE expression in male runners after a 7‐day stay at 1800 m altitude. Notably, Mckay et al. (2024) investigated athletes in lower altitude, and found no changes in EPO levels, which may have mitigated the following increase in ERFE. In the present study, a marked increase in EPO following the 6‐day LHTL was found, and therefore an insufficient erythropoietic drive was unlikely to limit the increase in ERFE. It should be noted, however, that the time course of our measurements may have prevented us from detecting possible transient changes in ERFE. Previously, Garvican‐Lewis et al. (2018) investigated ERFE and hepcidin responses to three‐week LHTL in endurance trained athletes (3000 m), and while they detected no significant changes in either marker, a spike in ERFE on the 3rd day of LHTL was observed, returning to baseline after 15 days of LHTL. Conversely, although Breenfeldt Andersen et al. (2021) reported that hypoxia‐induced elevation in ERFE declined over time, the authors found that ERFE remained significantly higher after three‐weeks of altitude residence when assessed at altitude. Płoszczyca et al. (2023), in turn, detected ERFE in trained male cyclists following 21 days of LHTL (2000 m) and reported decreased ERFE when measured immediately after the intervention. In the present study, ERFE remained unchanged after the 21‐day LHTL which is in line with the change reported by Garvican‐Lewis et al. (2018). It should be noted, however, that in the current study POST was conducted 1–2 days after returning to sea level, which may have resulted into ERFE and hepcidin to already return to pre‐altitude levels during the days spent at sea level. The above studies have all differences in time course of measurements as well as the type of hypoxic exposure (LHTL (Garvican‐Lewis et al., 2018; Płoszczyca et al., 2023) versus natural altitude (Breenfeldt Andersen et al., 2021; Mckay et al., 2024)), which may contribute to the inconsistent findings and limits direct comparisons. Additionally, these studies have largely varied with the participants' baseline iron levels and iron supplementation practices. In contrast to the present study, Breenfeldt Andersen et al. (2021) and Mckay et al. (2024) both investigated iron supplementing athletes with higher baseline ferritin levels (avg. ~70–125 μg·L^−1^), while Płoszczyca et al. (2023) neither assessed baseline ferritin levels nor had their participants supplementing iron. Due to these differences in the time course of blood sampling, hypoxic exposure, iron status, and study population, future research is required to clarify whether moderate hypoxia provides sufficient hypoxic stimulus to elevate ERFE levels to a degree that would make it a reliable and practical biomarker for monitoring iron demand in athletes.

Given that the LHTL did not elicit changes in ERFE, a hypoxic regulator of hepcidin, it was expected that hepcidin concentrations would remain unchanged. It is important to note, however, that the regulation of hepcidin is not solely connected to hypoxia‐induced changes in ERFE; it is largely affected by basal iron levels. Previously, in a normoxic condition, reduced resting and post‐exercise hepcidin have been observed in iron‐depleted athletes (Peeling et al., 2014). In the present study, five participants in INT exhibited stage 1 iron deficiency (Peeling et al., 2007) and the correlation analysis revealed lower baseline ferritin to be associated with a larger decrease in hepcidin from Day 0 to Day 6. This finding may imply that the additional iron demand placed on athletes with low iron stores when exposed to altitude can lead to compensatory reduction in hepcidin in the initial days of hypoxic exposure to support increased iron absorption and recycling. In this instance, we recommend individuals with low iron levels to supplement with iron to possibly capitalize on this reduction in hepcidin to prevent any declines in iron status that could adversely impact positive hematological adaptations with hypoxic exposure.

### 4.2 Routine iron markers

In addition to iron regulation, this study detected changes in routinely assessed iron markers. As mentioned in the previous section, serum ferritin reflects changes in iron stores and is often assessed prior to and during hypoxic training periods due to its possible influence on Hb_mass_ adaptations (Govus et al., 2015; Nummela et al., 2021). In the present study, we observed a significant reduction in serum ferritin from Day 0 to Day 21, but the change was similar across groups. The small reduction in ferritin levels in both groups may have been due to a negative iron balance resulting from iron loss with exercise exceeding iron intake. This negative iron balance can lead to mobilization of iron from internal iron stores, which overtime, can be detected as reduced serum ferritin (Pedlar et al., 2018). While in INT, the hypoxia‐induced increase in iron demand may have set an additional burden on body iron stores, sufficient iron intake via supplemental iron and/or nutritional sources may have mitigated the reduction in ferritin and led to the unchanged ferritin at group level.

Although no changes were detected in ferritin levels following the first 6 days of LHTL in INT, the correlation analysis revealed that a smaller reduction in ferritin from Day 0 to Day 6 was associated with higher Hb_mass_ response following the 21‐day LHTL. This observation may suggest that maintaining iron levels early during hypoxic exposure could contribute to achieving Hb_mass_ adaptations. Although iron supplementation did not appear to affect changes in ferritin levels, dietary iron intake was not monitored. However, depending on the participants' habitual dietary intake, adequate intake of nutritional iron sources may have reduced iron mobilization from internal iron stores during the period of increased iron demand (Govus et al., 2015; Koivisto‐Mørk et al., 2021). As such, in line with previous research, we recommend athletes to ensure sufficient iron intake when exposed to altitude both from nutritional and supplemental sources to prevent early reductions in iron stores and to potentially support hematological adaptations (Stellingwerff et al., 2019).

Of the measured iron demand markers transferrin, TSAT, and sTfR, only sTfR was observed to increase by 13.9 ± 16.4% from Day 0 to Day 21 in INT. The change aligns with that of previous studies reporting progressively increasing sTfR during and immediately after prolonged hypoxia, and suggests that sTfR reflects ongoing or recent increase in iron demand even after EPO response is blunted (Robach et al., 2004; Robertson et al., 2010). While transferrin remained unchanged in the current study, a positive correlation was found between Day 0 and Day 21 changes in transferrin and Hb_mass_ in INT. This finding implies a link between increased iron transport dynamics and erythropoietic response, where an increase in iron demand can contribute to an increased use of iron, which subsequently may lead to an increase in Hb_mass_. No changes were detected in any of the routine iron markers from Day 0 to Day 6 and these changes were not associated with changes in Hb_mass_. As such, our results suggest that similar to hepcidin and ERFE, the routinely assessed iron markers may not be sensitive to detect short‐term alterations in iron homeostasis during LHTL periods.

Taken together, our findings suggest that no single iron biomarker consistently reflects changes in iron demand and regulation during 6 or 21 days of LHTL, thereby limiting their utility to predict hypoxia‐induced changes in Hb_mass_ in female endurance athletes. Instead, we observed that pre‐hypoxic iron levels may influence the immediate changes in iron regulation, and the changes observed might reflect increased iron demand and hence increase iron absorption and recycling. Therefore, as per studies done in normoxic conditions, it would seem that baseline iron levels may be a stronger predictor of changes in iron demand at hypoxia compared to hepcidin levels or its hypoxic regulator. While factors such as hypoxia may regulate iron demand, detecting the time point for the change in iron demand and hence regulation would be challenging in studies in the field (Galetti et al., 2021; Peeling et al., 2014). Nevertheless, a comprehensive assessment of markers of iron stores, demand, and regulation before and after hypoxia, combined with a review of nutritional intake and possibly exercise performance, could provide insights into the effectiveness of any given hypoxic dose that might provide hematological adaptations for the athlete.

### 4.3 Limitations

This study has several limitations. Firstly, we did not monitor menstrual cycle phase or control the use of hormonal contraceptives and iron intake. These factors are known to influence iron regulation and thereby could have affected the results of the present study (Badenhorst et al., 2022). To our knowledge, however, existing literature investigating the impact of hormonal contraception on iron markers has only focused on the use of combined hormonal methods, whereas in the present study, progestin‐only contraception was the sole form used. In our study, the use of hormonal contraceptives as well as iron supplementation did not appear to influence changes in any of the iron markers, but the possible impact of variations in menstrual cycle phase cannot be excluded. In addition, this study did not assess platelet‐derived growth factor‐BB, which has been observed to influence hepcidin expression in hypoxia along with ERFE and basal iron levels (Sonnweber et al., 2014). Therefore, it is possible that the platelet‐derived growth factor‐BB also contributed to stabilization of hepcidin expression throughout the intervention in INT and should be considered in future investigations.

Limitations also exist in the assessment of iron markers. Despite the adherence to the manufacturer's instructions, the observed ERFE levels were higher than those reported in previous studies (Breenfeldt Andersen et al., 2021; Mckay et al., 2024; Płoszczyca et al., 2023). However, all the samples were analyzed under identical conditions using the same assay kit and the results exhibited strong alignment on the standard curve. Thereby the results indicate that the observed variations in ERFE levels can be estimated with high reliability. There were also limitations in the Hb_mass_ assessment, as we did not use duplicate measurements as has been recommended for athletes (Hauser et al., 2017). Although we aimed to reduce the measurement error via familiarization to the breathing protocol, clear instructions, and by collecting multiple blood samples from a fingertip, performing only a single rebreathing measurement may have influenced the precision of the Hb_mass_ assessment and thus the ability to detect hypoxia‐induced Hb_mass_ adaptations.

## 5 CONCLUSIONS

The results of the present study suggest that, in isolation, hepcidin and ERFE may not provide additional information regarding changes in iron demand and regulation following 6 or 21 days of LHTL compared to routine iron markers. The associations between Hb_mass_ adaptations and changes in serum ferritin, in turn, suggest that maintaining ferritin levels in hypoxia may support achieving the hematological adaptations and thus the desired improvements in performance. As such, our results align with the current practical recommendations of dietary and supplementary iron use prior to and during prolonged hypoxic training camps (Stellingwerff et al., 2019).

## AUTHOR CONTRIBUTIONS

TK, JKI, VL, OK, and RSM conceptualized the research. TK and OK were involved in data collection and curation. TK performed the formal analysis. TK and VL were involved in the funding acquisition. JKI, VL, and RSM were involved in supervision. TK wrote the original draft of the manuscript and prepared figures. JKI, VL, CB, OK, and RSM reviewed and edited the manuscript. TK, JKI, VL, CB, OK, and RSM approved the final version of the manuscript.

## FUNDING INFORMATION

This study was supported by the Finnish Ministry of Education and Culture (OKM/2/626/2023), Seppo Säynäjäkangas Science Foundation (12/2023), and the Kainuu Regional Fund of the Finnish Cultural Foundation (5/2024).

## CONFLICT OF INTEREST STATEMENT

The authors declare no conflicts of interest, financial or otherwise.

## ETHICS STATEMENT

The ethical committee of University of Jyväskylä approved the study (29/13.00.04.00/2021), and all the measurements were conducted in accordance with the Declaration of Helsinki except for pre‐registration in a database.

## Data Availability

The datasets used and/or analyzed during the current study are available from the corresponding author on reasonable request.
